# Knowledge graph embedding for predicting and analyzing microbial interactions

**DOI:** 10.1038/s41598-025-27591-9

**Published:** 2025-11-21

**Authors:** Mohammed Khatbane, Cécile Mangavel, Frédéric Borges, Sabeur Aridhi, Yannick Toussaint

**Affiliations:** 1 Université de Lorraine, CNRS, LORIA, 54000 Nancy, France; 2 Université de Lorraine, LIBio, 54000 Nancy, France

**Keywords:** Knowledge graph embedding, Microbial interactions, Machine learning, Microbial ecology, Microbial ecology, Machine learning

## Abstract

Interactions between microorganisms play a major role in shaping the structure and function of microbial communities, yet their prediction remains a challenge in microbial ecology. While currently available machine learning methods have shown promising performance, they often rely on extensive input features that are obtained from labor-intensive experiments. Here, we propose a new framework to predict pairwise interactions that minimizes the need for in vitro experimentation. Our approach is based on knowledge graph embedding, which learns the representation of microorganisms and their interactions in an embedding space. Using a dataset of interactions between 20 soil bacterial strains cocultured in 40 different carbon source environments, we demonstrate the effectiveness of our framework in accurately predicting pairwise interactions. Notably, we show that our model can predict interactions involving strains with missing culture data. We additionally show that the obtained embeddings can reveal similarities between carbon source environments, enabling the prediction of interactions in one environment based on the outcomes in a similar environment between the same pair of microorganisms. Furthermore, our approach allows the design of a recommendation system that can be used to guide microbial community engineering. These findings demonstrate that knowledge graph embedding is a promising modeling strategy in microbial ecology.

## Introduction

Microorganisms have a crucial role in all ecosystems^1^, and particularly influence human health, agri-food systems, and climate change^2–4^. In microbial communities, microorganisms establish interactions with each other at intra- and interspecies levels, as well as with their hosts^5,6^. They exhibit various interaction types including competition, amensalism, parasitism, commensalism, neutralism and mutualism^7^. For example, mutualistic interactions (cooperation) occur when the fitness of individuals from both species increases^8^. In this case, the species benefit from the presence of the other species. In contrast, competitive interactions can be classified into exploitative (passive) and interference (active) competitions^9,10^. Exploitative competition is characterized by a rapid use of the limiting resource, resulting in the exclusion of less fit competitors. On the other hand, interference competition is defined as a direct antagonism in which one species inhibits the growth of competitors by producing antimicrobial substances^11,12^. This complexity makes the analysis and engineering of microbial communities a challenging task. Nevertheless, as interspecies interactions shape the community structure and function^13,14^, their analysis would allow us to better understand the dynamics of microbial communities, and thus improve the control of the structure and thereby the function of these communities.

Quantifying the interactions between microbial species often involves comparing how species grow or behave alone versus when they are together^15^. Particularly, modeling approaches to microbial community structure have mainly focused on interactions between pairs of species^16–18^. Recent studies have shown that the outcome of these pairwise interactions can be used for predicting the structure of multi-species communities^19,20^. However, measuring all interactions is experimentally challenging even for low-diversity communities. Therefore, developing advanced approaches for predicting them is needed.

Recently, machine learning has emerged as a powerful tool in microbiology^21,22^. Various machine learning approaches have been applied to predict pairwise microbial interactions, including tree-based models, deep learning, K nearest neighbors, and logistic regression models. For example, the effectiveness of the XGBoost model in predicting the strength and type of interspecies interactions based on features like monoculture growth and species’ phylogeny has been demonstrated^23^. Similarly, Random Forest has been successfully used to predict microbial interactions based on trait-level descriptions^24^. In addition, deep learning-based techniques have been used to effectively predicting microbial interactions using spatial patterns of microbes^25^, as well as to predict microbiome composition^26^. More recently, graph-based techniques have provided novel insight into model microbial communities. Specifically, Graph Neural Networks (GNNs) have shown promising performance in predicting the composition of bacterial communities from their genomes^27^. Despite their success, existing methods for predicting pairwise interactions present certain limitations. For instance, they rely on extensive input features that are challenging to collect experimentally such as metabolic profile data across all environments considered in the analysis. Additionally, they treat each interaction as an independent instance, ignoring the interconnected nature of interactions in microbial communities.

To overcome these limitations, knowledge graph embedding (KGE) offers a promising alternative to efficiently model microbial interactions by leveraging the flexibility of knowledge graph (KG) in representing the data as triples of a subject, predicate, and object, and learning their representation in an embedding space. KGs are structured representations of real world information^28^. Formally, a KG can be defined as $\mathscr {G} = (\mathscr {E}, \mathscr {R}, \mathscr {T})$, where $\mathscr {E}$ denotes a set of nodes (known as *entities*), $\mathscr {R}$ represents a set of relations and $\mathscr {T}\subset \mathscr {E} \times \mathscr {R} \times \mathscr {E}$ a set of triples (facts) of the form (*h*, *r*, *t*) where the nodes $h, t \in \mathscr {E}$ are connected by the relation $r \in \mathscr {R}$ indicating a directed link from *h* to *t*^29^, *e.g.*, (*Escherichia coli*, *canMetabolize*, *Lactose*). KGs are inherently incomplete^30^, and one of the main challenges consists of predicting unobserved links between entities in order to complete the graph. This task is known as knowledge graph completion or link-prediction (LP) problem^31^. To address this challenge, various methods have been proposed in the literature and KGE has become popular in recent years. Briefly, KGE is a machine learning approach designed to learn the representation of entities and relations in a low-dimensional vector space, while ensuring the preservation of the KG’s structure and capturing the semantic meaning of entities and relations^29^.

To date, the application of KGE for predicting microbe-microbe interactions remains unexplored. Thanks to their ability to capture the multi-relational structure of graphs, KGE techniques could provide an effective approach for predicting microbial interactions without relying on extensive and time-consuming experimental features. Yet these methods have already proven effective in many related fields including antibiotic gene resistance discovery^32^, predicting side effects of drug combinations^33^, drug–target interactions^34^, drug repurposing for COVID-19^35^ and, more recently, the prediction of microbe-host interactions^36^.

In this study, we explore the use of KGE techniques for predicting microbial interactions and introduce a novel framework called KGEMI (Knowledge Graph Embedding for Microbial Interactions). KGEMI is capable of performing multiple tasks related to modeling and analyzing microbial interactions. First, we propose a new approach for predicting the interactions between microorganisms using a KGE framework and demonstrate its effectiveness. Second, the analysis of carbon source environment embeddings allows us to develop an environment-based decision rule to infer the interactions using the similarities between environments. Third, the obtained embeddings enable us to design an effective method for predicting interactions involving strains for which cultivation data is missing. Lastly, we propose a KGE-based recommendation system to guide microbial community engineering. Here, we use the term interaction in a phenomenological sense, referring to the effect of one strain on another’s growth in coculture compared to monoculture. This usage follows the original experimental design by Kehe et al.^37^ and does not imply any direct mechanistic interpretation. Rather than describing ecological processes such as chemical inhibition or mutualistic signaling, the term refers to observed growth differences under controlled conditions.

## Methods

### Knowledge graph construction

Pairwise interactions between microorganisms were modeled using a KG in which the entities (nodes) represent microbial strains, and the interaction types along with the carbon environment define the relationship (edge) between these strains.

In order to construct a KG, we used a dataset provided by Kehe et al.^37^ and Nestor et al.^23^. It contains microbial interaction data between 20 soil bacterial species cocultured in 40 different media differing by the carbon source. Given two cocultured strains, $S_1$ and $S_2$, the authors evaluated the effect of the competitor strain $S_2$ on the focus strain $S_1$ using the following formula:

1 $$\begin{aligned} \text {E}_{S_{2} \rightarrow S_{1}} = \log \left( \frac{\text {Coculture}(S_{1} | S_{2})}{\text {Monoculture}(S_{1})} \right) ; \end{aligned}$$

where $\text {Coculture}(S_1 | S_2)$ represents the growth yield of $S_1$ in the presence of $S_2$, and $\text {Monoculture}(S_1)$ is the growth yield of $S_1$ in monoculture in a given carbon source environment. $\text {E}_{S_2 \rightarrow S_1}$ quantifies the impact of the competitor $S_2$ on the growth of $S_1$ by comparing its growth yields in the coculture and the monoculture conditions. The resulting effects were then discretized into three categories depending on the effect value: Positive, Negative and Neutral. A positive value indicates that $S_2$ enhances the growth of $S_1$, a negative value indicates that $S_2$ inhibits the growth of $S_1$ and 0 signifies no detected effect or neutral interaction. Subsequently, for each interaction where $S_2$ affects $S_1$ in a specific carbon environment *e*, triples were constructed in the graph as ($S_2$, *Positive in* *e*, $S_1$), indicating a positive effect of strain $S_2$ on $S_1$ within the specific environment *e*, and similarly for negative and neutral interactions. Although the original dataset includes coculture measurements for most pairwise combinations of 20 strains across 40 carbon source environments, some interactions are missing, resulting in an incomplete knowledge graph. The description of all 20 microbial strains used in this study are provided in Supplementary Table S1.

### Knowledge graph embedding

In knowledge graph embedding (KGE), an embedding is a vector that represents a node (e.g., microbial strain) or a relation (e.g., interaction type within a specific carbon source environment). The goal of KGE is to learn these vector representations so that the structure of the graph is captured in a way that allows the prediction of missing links (e.g., interactions). In this study, each microbial strain is represented as a node, and interactions between strains are represented as directed edges (relations). Each relation corresponds to both the interaction type (Positive, Negative, or Neutral) and the carbon source environment in which the interaction occurs. This means that for each environment, we define up to three distinct relations, one for each interaction type, depending on the presence of that interaction type in the environment. Depending on the KGE model, each entity and relation is generally represented by a vector embedding in $\mathbb {R}^d$, where *d* is the embedding dimension. Following^38^, we use boldface characters to denote these vectors: $\textbf{h}, \textbf{r}, \textbf{t}$ for the head (sender), relation, and the tail (receiver), respectively. Since a relation combines both interaction type and environment, its embedding encodes both. However, when we need to represent an environment alone (for example, to calculate similarities between environments), we construct its embedding by concatenating all relation embeddings associated with that environment.

**Fig. 1:**
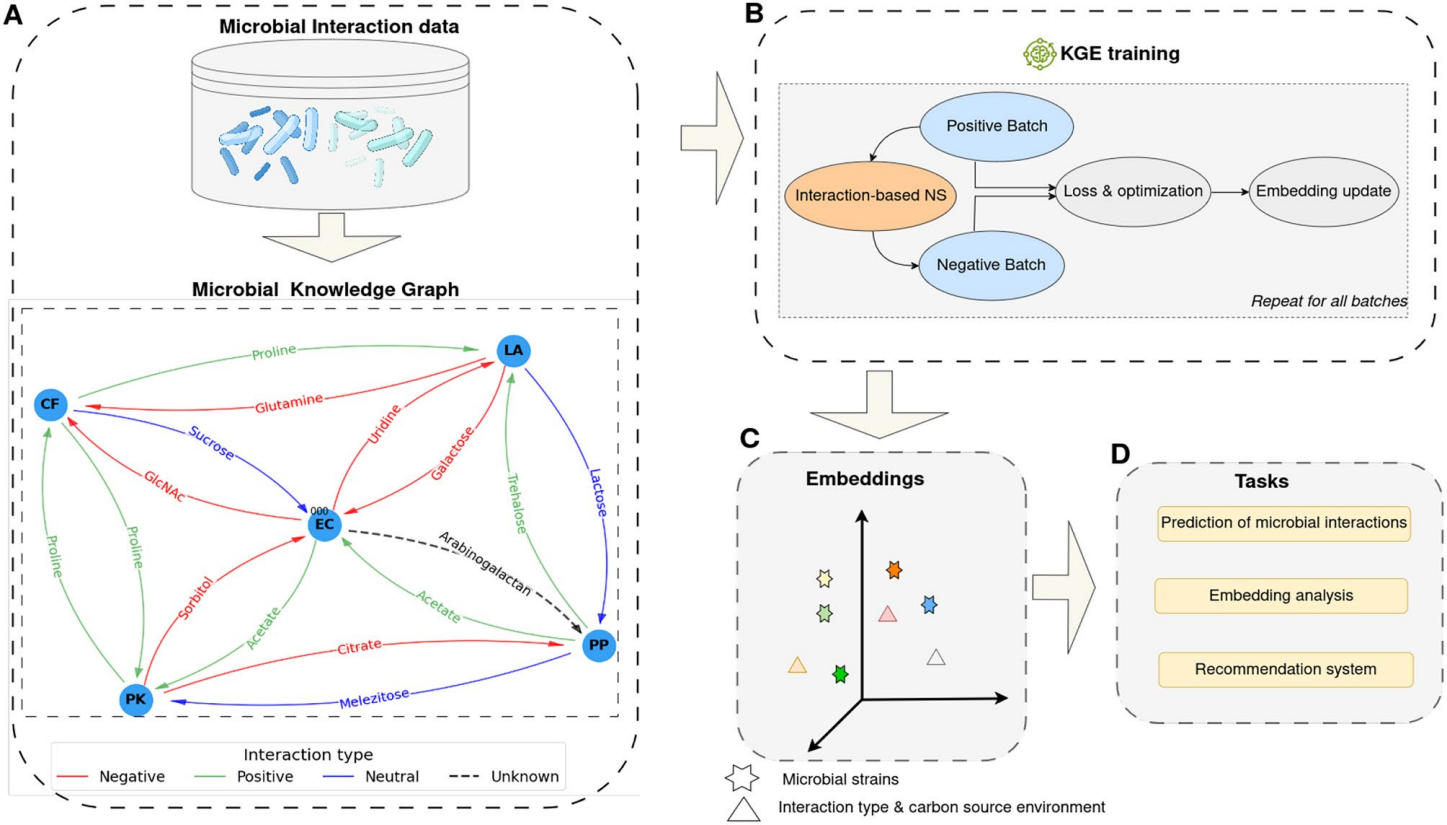
KGEMI architecture. The framework first transforms microbial data into a structured knowledge graph (**A**), where the nodes (*entities*) represent microbial species and the relations between them are characterized by both the interaction type (*positive*, *negative* or *neutral*) and the environment in which the interaction occurs (*e.g.*, glucose, lactose). The resulting knowledge graph is used to train a KGE model (**B**), which generates the embeddings for microorganisms and their relationships (**C**). These embeddings are then used for various tasks (**D**) such as the prediction of interactions, microbial interaction analysis and the design of a recommendation system.

Let $\mathscr {E}$ be a set of entities representing the strains, and $\mathscr {R}$ the set of relations between them. Each relation $r = (I,e) \in \mathscr {R}$ consists of an interaction type *I* and a carbon source environment *e*. For example, if *Escherichia coli* inhibits the growth of *Lelliottia amnigena* in a uridine environment, both strains are represented as nodes with a directed link from *Escherichia coli* to *Lelliottia amnigena* labeled with the relation *negative in uridine* (Fig. 1).

To learn the representation of all entities and relations in order to predict the interaction type $I \in \{\text {Positive, Negative, Neutral} \}$, three KGE models were tested, namely, TransE^38^, DistMult^39^ and SimplE^40^. After constructing the KG, it was first split into $90\%$, $5\%$ and $5\%$ for training, validation and test sets respectively. Given two entities $h, t\in \mathscr {E}$ and a relation $r\in \mathscr {R}$, for each model a scoring function $\varphi : \mathscr {E} \times \mathscr {R} \times \mathscr {E} \rightarrow \mathbb {R}$ is defined to measure the plausibility of the triple (*h*, *r*, *t*). The learning process of the embeddings consists of maximizing the score $\varphi$ for observed triples (facts) and minimizing it for the false triples that are generated by corrupting the observed facts.

In this study, the embeddings for nodes (microbial strains) and relations (interaction type–environment pairs) are real-valued vectors of a common length $d$, a hyperparameter independent of $\mathscr {E}$ and $\mathscr {R}$ that is tuned for each model. The coordinates of these vectors are first randomly initialized and then optimized during the training so that the model assigns higher scores to observed triples and lower scores to corrupted ones.

For example, if strain A (sender) has a negative effect on strain B (receiver) in a *glucose* environment, this observation is represented as the triple $(A, r_{\text {negative, glucose}}, B)$. During training, a corresponding false triple can be generated, e.g., $(A, r_{\text {positive, glucose}}, B)$, which represents the same pair of strains but with an incorrect interaction type. The embedding vectors $\textbf{h}_A = (h_1, \dots , h_d)$, $\textbf{t}_B = (t_1, \dots , t_d)$, $\textbf{r}_{\text {negative, glucose}} = (r_1, \dots , r_d)$, and $\textbf{r}_{\text {positive, glucose}} = (r'_1, \dots , r'_d) \in \mathbb {R}^d$ are adjusted during training so that:

2 $$\begin{aligned} {\varphi (A, r_{\text {negative, glucose}}, B) > \varphi (A, r_{\text {positive, glucose}}, B).} \end{aligned}$$

It is important to emphasize that the relation embedding $\textbf{r}_{\text {negative, glucose}}$ is *global*, meaning that it is shared across all triples that correspond to negative interactions occurring in the glucose environment, regardless of the specific strain pair. In contrast, $\textbf{h}_A$ and $\textbf{t}_B$ are unique to each microbial strain and capture their overall interaction tendencies across environments. Each coordinate of these embeddings has no direct biological meaning by itself, but together they capture statistical patterns in the observed interaction data, allowing the model to generalize and predict new plausible interactions.

In TransE^38^, which was the first geometrical KGE model^28^, the relation *r* in a triple (*h*, *r*, *t*) is interpreted as a translation in the embedding space, meaning that ${\textbf {h}}, {\textbf {r}}$ and ${\textbf {t}} \in \mathbb {R}^d$ are learned such that ${\textbf {h}} + {\textbf {r}} \approx {\textbf {t}}$. The scoring function is defined as:

3 $$\begin{aligned} \varphi (h, r, t) = - \Vert \textbf{h} + \textbf{r} - \textbf{t}\Vert ; \end{aligned}$$

where $\Vert \cdot \Vert$ denotes the L1 or L2 norm. A high score indicates that the vectors ${\textbf {h + r}}$ and ${\textbf {t}}$ are close in the embedding space $\mathbb {R}^d$.

DistMult^39^ is a semantic matching-based model that uses a simplified bilinear scoring function to assess the plausibility of triples, where the score is computed as:

4 $$\begin{aligned} \varphi (h, r, t) = \langle \textbf{h}, \textbf{r}, \textbf{t} \rangle = \sum _{i=1}^{d} {\textbf {h}}_i \cdot {\textbf {r}}_i \cdot {\textbf {t}}_i \end{aligned}$$

SimplE^40^ models each entity by two embeddings to capture both its head and tail behaviors, as well as two embeddings associated with the relation, one representing the relation itself and the other for its inverse direction. Its scoring function is given by:

5 $$\begin{aligned} \varphi (h, r, t) = \frac{1}{2}(\langle \textbf{h}_h, \textbf{r}, \textbf{t}_t \rangle + \langle \textbf{h}_t, \textbf{r}^{-1}, \textbf{t}_h \rangle ); \end{aligned}$$

where $\textbf{h}_h$ and $\textbf{h}_t$ are the head and tail embeddings of entity *h*, $\textbf{t}_h$ and $\textbf{t}_t$ are the head and tail embeddings of entity *t*, $\textbf{r}$ is the embedding of relation *r*, and $\textbf{r}^{-1}$ is the embedding of the inverse relation.

All these models capture the representation of each node in its context within the KG, meaning that microorganisms that share similar interactions will have closer vector representations than those with dissimilar interactions.

The embeddings are learned by minimizing some loss function over the training set. Here, we used two loss functions that are commonly used in the literature: *Pairwise ranking loss* (Eq. 6) and *Pointwise loss* (Eq. 7).

6 $$\begin{aligned} \mathscr {L}_{\text {pairwise}} = \sum _{(h,r,t) \in \mathscr {D}^+} \sum _{(h',r',t') \in \mathscr {D}^-} \max (0, \gamma - \varphi (h,r,t) + \varphi (h',r',t')) \end{aligned}$$

7 $$\begin{aligned} \mathscr {L}_{\text {logistic}} = \sum _{(h,r,t, l)\in \mathscr {D}^{+} \cup \mathscr {D}^{-}} \log (1 + \exp (-l \times \varphi (h,r,t))); \end{aligned}$$

where $\mathscr {D}^+$ is the set of observed triples in the training set, $\mathscr {D}^-$ a set of false triples (generated by corrupting the observed triples), $\gamma > 0$ a margin hyperparameter and *l* is the label of the triple: $l = +1$ if the triple is observed and $-1$ otherwise.

The training of the models was performed in mini-batch mode using SGD (stochastic gradient descent) as in^38–42^. The embeddings of the entities and relations were first randomly initialized. Then each batch $\mathscr {B}^{+}$ of triples was sampled from the training set, with which a batch $\mathscr {B}^{-}$ of false triples was generated by corrupting the head, tail or the relation of triples in $\mathscr {B}^{+}$ depending on the chosen negative sampling (NS) method. Subsequently, the loss function is calculated over the batch $\mathscr {B} = \mathscr {B}^+ \cup \mathscr {B}^-$ followed by the calculation of the gradients and finally the embeddings are updated using SGD. Here, we used a Pytorch implementation of SimplE for training and evaluating the models. Following^43^, the pairwise ranking loss was used for the TransE model and pointwise loss for the SimplE and DistMult models. The hyperparameters were tuned using grid search over the validation set for each model. For more consistent comparison, we retrained and reported the results of 100 experiments for each model.

### Interaction-based negative sampling

Existing NS methods typically generate false triples by corrupting either the head, tail or both components of observed triples. However, in dense or nearly complete knowledge graphs, such as ours, where nearly all strain pairs are observed across environments, this strategy may occasionally produce triples that correspond to true interactions (false negatives). Although classical NS was used as a baseline in our experiments, we did not filter out such cases, which is common in standard KGE training pipelines but can introduce noise in dense graphs.

Since we are interested in predicting the interaction type, which is part of the relation, we developed a simple NS approach that focuses on the relation part of the triple rather than the head or tail.

Let $\mathscr {I}$ represent the set of interaction types (positive, negative and neutral) and (*h*, *r*, *t*) a triple in the KG, where *h* is the sender strain, *t* the receiver and $r = (I,e)$ is the relation composed of the interaction type $I \in \mathscr {I}$ and the environment *e*. Our approach generates false triples by corrupting the relation *r*, particularly by replacing *I* with a different interaction type $I' \in \mathscr {I}\setminus {I}$ while keeping the environment unchanged. In other words, the false triple associated with (*h*, *r*, *t*) is simply $(h, r', t)$ where $r' = (I', e)$.

For example, if strain $S_1$ has a positive effect on strain $S_2$ in the environment *e*, then this information is represented in the KG as the triple $(S_1, r, S_2)$ with $r = (\text {Positive}, e)$. A corresponding false triple could be generated as $(S_1, r', S_2)$ with $r' = (\text {Negative}, e)$ or $r' = (\text {Neutral}, e)$. Since there are three interaction types, the selection of the false interaction is performed randomly from the remaining two options.

### Evaluation metrics

To evaluate the performance of the models, the following metrics were used: accuracy , recall , precision and F1-score. These measures are defined as follows:

8 $$\begin{aligned} \text {Accuracy} = \frac{TP + TN}{TP + TN + FP + FN} \end{aligned}$$

9 $$\begin{aligned} \text {Recall} = \frac{TP}{TP + FN} \end{aligned}$$

10 $$\begin{aligned} \text {Precision} = \frac{TP}{TP + FP} \end{aligned}$$

11 $$\begin{aligned} \text {F1-score} = 2 \times \frac{\text {Precision} \times \text {Recall}}{\text {Precision} + \text {Recall}} \end{aligned}$$

Where *TP* denotes true positives, *TN* true negatives, *FP* false positives and *FN* false negatives. In addition to these metrics, confusion matrices were also reported to provide more detailed information about the classification performance.

Moreover, considering the problem as a link prediction task, we used the Mean Reciprocal Rank (MRR) and Hits@k as link prediction metrics. These evaluation metrics are based on the rank of the triples in the test set $\mathscr {G}_{\text {test}}$. For each triple $(h,r,t) \in \mathscr {G}_{\text {test}}$, the head-based rank $\text {rank}_{(h,r,t)}^{h}$ is determined by comparing the score of (*h*, *r*, *t*) against all triples in the form $(h', r, t),\ h'\in \mathscr {E}$. Similarly, the tail-based rank $\text {rank}_{(h,r,t)}^{t}$ is calculated based on the score of triples in the form $(h, r, t'),\ t'\in \mathscr {E}$. The MRR (Eq. 12) represents the average of the reciprocal ranks, and Hits@k (Eq. 13) measures the proportion of ranks within the top *k* positions.

12 $$\begin{aligned} \text {MRR} = \frac{1}{2 N} \sum _{(h,r,t)\in \mathscr {G}_{\text {test}}} \frac{1}{\text {rank}_{(h,r,t)}^{h}} + \frac{1}{\text {rank}_{(h,r,t)}^{t}} \end{aligned}$$

13 $$\begin{aligned} \text {Hits@k} = \frac{|E^h| + |E^t|}{2 N}; \end{aligned}$$

where:

$$\begin{aligned} E^h = \{(h,r,t)\in \mathscr {G}_{\text {test}};\ \text {rank}_{(h,r,t)}^{h} \leqslant k\},\ E^t = \{(h,r,t)\in \mathscr {G}_{\text {test}};\ \text {rank}_{(h,r,t)}^{t} \leqslant k\} \end{aligned}$$

and $$N = |\mathscr {G}_{\text {test}}|$$.

### Prediction strategy for uncultured microbial strains

**Fig. 2:**
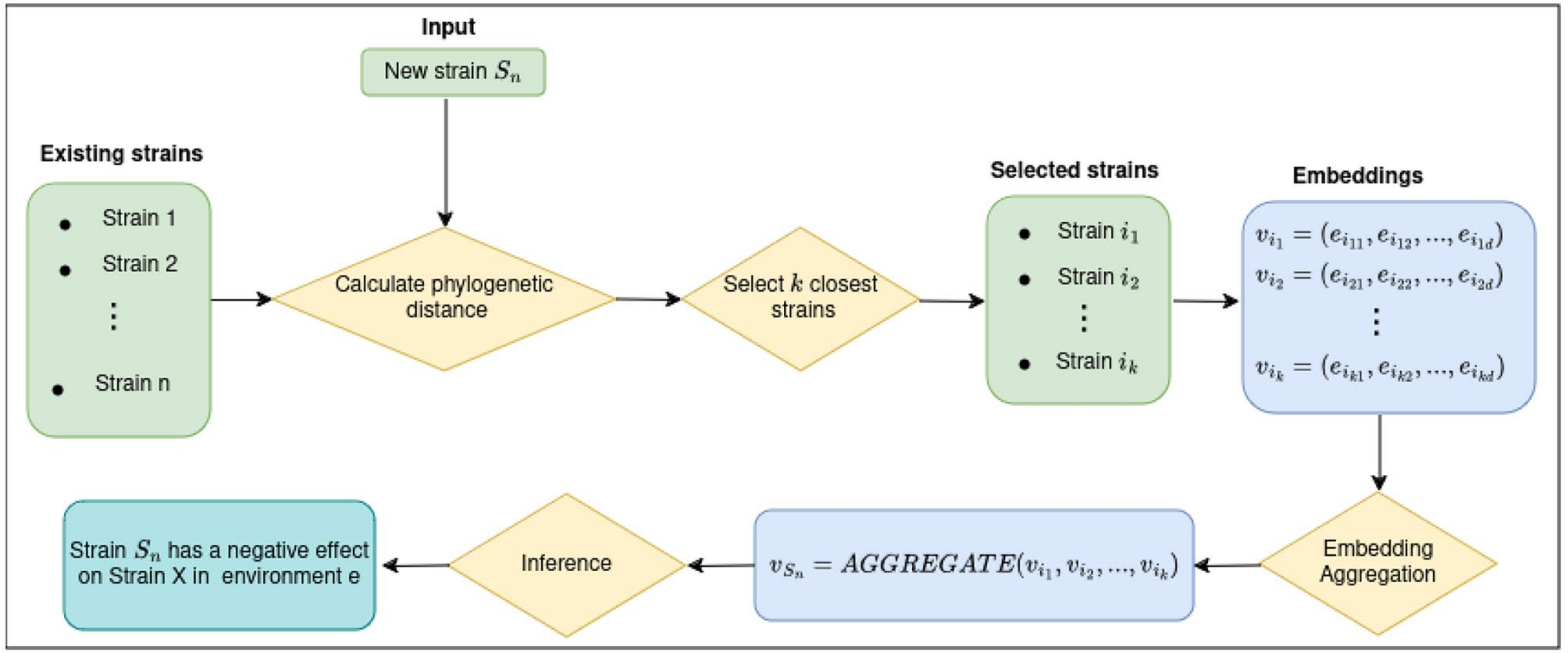
Prediction process for new strains. Existing strains refer to those that are present in the dataset, with embeddings $v_{i_j}$ obtained from the trained model. The process identifies strains phylogenetically related to the new strain and aggregates their embeddings using an AGGREGATE function, resulting in a synthetic embedding that enables the predictions for the new strain.

Since KGE models are not designed to make prediction for nodes that were unseen during the training, we developed a new strategy allowing the prediction for strains that are not in the KG. Given a carbon source environment *e*, a strain $S \in \mathscr {E}$, and a new strain $S_n \notin \mathscr {E}$, the goal is to predict the outcome of the interaction between $S_n$ and *S* within the environment *e*. The main idea consists of making predictions for $S_n$ based on the embeddings of strains in the graph that are close to the new strain $S_n$ phylogenetically. To quantify phylogenetic similarity, we used features provided by Nestor et al.^23^, who computed a phylogenetic distance matrix based on 16S rRNA gene sequences and extracted features using PCA that capture over $95\%$ of the variance. Since the original distance matrix was not publicly available, we computed pairwise Euclidean distances between strains in the PCA feature space, which we refer to as phylogenetic distances. As described in Fig. 2, after calculating the phylogenetic distances based on phylogenetic features provided by Nestor et al.^23^, between the target $S_n$ and all strains $S \in \mathscr {E}$, the *k* strains that are the closest to the target were selected. Subsequently, the embeddings of these strains were aggregated using an aggregation function to create one single vector representation that was considered a synthetic embedding of the new strain. Here, we used the weighted average (Eq. 14) as an aggregation function as it makes the closest strains contribute more than the others. The synthetic embedding was then used to calculate the score and therefore to make predictions.

14 $$\begin{aligned} \textbf{v}_{S_n} = \sum _{j = 1}^k w_j \textbf{v}_{i_j}; \end{aligned}$$

where $i_1, i_2, \dots , i_k$ denote the indices of the *k* phylogenetically closest strains selected from the set of existing strains $\mathscr {E}$. $\textbf{v}_{i_j}$ represents the embedding of the $j^{th}$ selected strain, and $w_j$ is the corresponding weight. The weights were calculated and normalized to give more importance to the strains that are close to the target, compared to the others:

15 $$\begin{aligned} w_j = \frac{1}{ 1 + d_j}; \end{aligned}$$

where $d_j$ is the distance between the strain $S_{i_j}$ and the target. Hence, if this distance is relatively small, $w_j$ will make the associated strains contribute more to the calculation of the synthetic embedding.

### Environment-based prediction

To build a decision rule based on the similarities between environments, we first created a vector representation for each environment. Since an environment *e* can be involved in three relations $r_1 =$ ”*negative in* *e*”, $r_2=$ *”positive in* *e*” and $r_3=$ *”neutral in* *e*”, we constructed a vector $\textbf{v}_e$ representing the embedding of *e* by concatenating the embedding vectors of these three relations:

16 $$\begin{aligned} \textbf{v}_e = (\textbf{r}_1 | \textbf{r}_2 | \textbf{r}_3); \end{aligned}$$

where | denotes the concatenation operator, and $\textbf{r}_1, \textbf{r}_2, \textbf{r}_3$ are the learned embeddings of the relations $r_1, r_2,$ and $r_3$ respectively. To quantify the similarity between two environments $e_i$ and $e_j$, we computed the distance between their vector representations $\textbf{v}_{e_i}$ and $\textbf{v}_{e_j}$. Here, we tested two distance metrics: euclidean distance (Eq. 17) and cosine distance (Eq. 18):

17 $$\begin{aligned} d_1(e_i, e_j) = \Vert \textbf{v}_{e_i} - \textbf{v}_{e_j} \Vert _2 ; \end{aligned}$$

18 $$\begin{aligned} d_2(e_i, e_j) = 1 - \frac{\textbf{v}_{e_i} \textbf{v}_{e_j}^T}{\Vert \textbf{v}_{e_i}\Vert _2 \Vert \textbf{v}_{e_j}\Vert _2}; \end{aligned}$$

where $\Vert \cdot \Vert _2$ is the $L_2$ norm.

When at least one of the interaction types was missing for an environment in the dataset, the corresponding environment was excluded from the analysis. For each environment, a list of all other environments was generated, sorted by increasing similarity (i.e., decreasing distance). The interaction between two strains in the test set was predicted by copying the observed interaction between the same pair of strains in the most similar environment. When the interaction was missing for the most similar environment, the observed interaction within the second environment in the list was considered, and so on.

### KGE-based recommendation system

Let $\mathscr {I}$ be the set of the interaction types (positive, negative and neutral) and $S_t$ a target strain. To identify the best candidates most likely to impact the target strain with the interaction $I \in \mathscr {I}$ in a given carbon source environment *e*, the query was formulated as a triple: $(?, (I, e), S_t)$. For candidate strains $S_1, \dots , S_k$ , the scoring function of the trained model was calculated for each triple $(S_i, (I, e), S_t), i = 1, \dots , k$. The resulting scores were subsequently sorted in descending order, with lower ranks indicating better candidates. Formally, the recommended strain $S_{rec}$, top-ranked, was defined as:

19 $$\begin{aligned} S_{\text {rec}} = \arg \max _{i = 1, \dots , k }\varphi (S_i, r, S_t); \end{aligned}$$

where $\varphi$ is the scoring function and $r = (I, e)$. If the query was formulated to evaluate the inverse effect, *i.e.*, $(S_t, (I, e), ?)$, then the same process was applied, but with the scoring function adjusted to account for the reversed order of the query.

**Fig. 3:**
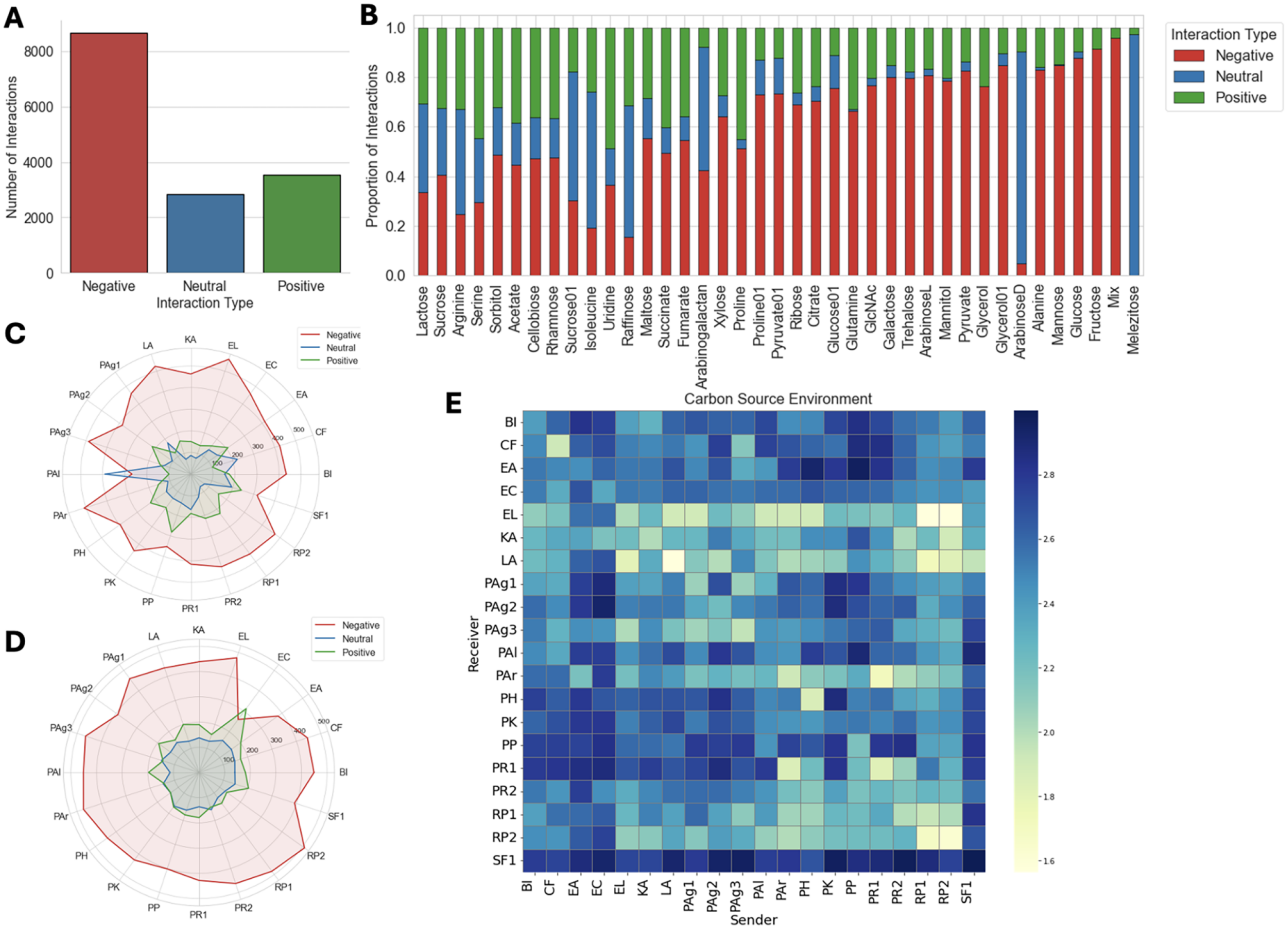
Overview of interaction class distributions across different contexts in the dataset. **A** Global distribution of interaction classes. **B** Class distribution within each individual environment, sorted in descending order of class entropy. **C** Global distribution of interaction classes when strains act as receivers. **D** Global distribution of interaction classes when strains act as senders. **E** Effective number of interaction types ($2^{H}$) for each pair of strains across all environments.

### XGBoost baseline

To provide a comparative baseline, we used an XGBoost classifier^44^. This model was previously applied to the same microbial interaction dataset used in this study by Nestor et al.^23^, and has shown strong performance in predicting pairwise interactions. The model was trained to predict the interaction type between two strains in a specific carbon source environment. Input features included the sender and receiver strain identifiers, as well as the carbon source environment. These categorical variables were encoded using one-hot encoding. The same training, validation, and test splits were used as in our KGE approach, and hyperparameter tuning was conducted on the validation set using grid search.

### Entropy calculation

To measure the imbalance of interaction types in the dataset, we calculated the entropy of their distribution using the following formula:

20 $$\begin{aligned} H = - \sum _{c \in \{-, 0, +\}} p(c) \log _2 p(c); \end{aligned}$$

where *p*(*c*) is the proportion of each interaction type. A lower entropy value indicates a stronger imbalance (i.e., one class dominates), while a higher entropy value reflects a more uniform distribution across the interaction types.

### Software implementation

All models were implemented using PyTorch (version 1.11.0, https://pytorch.org) for training and evaluation of the knowledge graph embedding techniques. The XGBoost classifier used for baseline comparisons was implemented using XGBoost (version 1.7.5, https://xgboost.ai). All experiments were conducted using Python 3.9.

## Results

### Dataset description

In this study, we used a dataset consisting of pairwise interactions between 20 soil bacterial strains cocultured in 40 different carbon source environments^23,37^. The effect of one bacterial strain on another was assessed using the log ratio of the growth yield in monoculture and coculture (Eq. 1). We first discretized this continuous effect into three interaction types: negative, positive and neutral, which resulted in $57.6 \%$ of negative interactions, $23.6 \%$ of positive and $18.9\%$ of neutral interactions. Figure 3 provides an overview of how interaction classes are distributed across the dataset and serves as a basis for designing different null models to assess how difficult it is to predict the interaction types using simple constraints. The global class imbalance (Fig. 3A), where negative interactions dominate, motivates a simple global majority baseline (Null Model A) that predicts the interaction type as the most frequent class (Negative). Figure 3B shows the distribution of interaction types across different carbon source environments, sorted in descending order of entropy. While some environments (e.g., Melezitose) are dominated by a single interaction type (e.g., Neutral) others (e.g., Lactose) show more balanced class distribution. This analysis motivated the environment-specific majority baseline (Null Model B), which predicts the interaction type as the most frequent class within each environment.

**Fig. 4:**
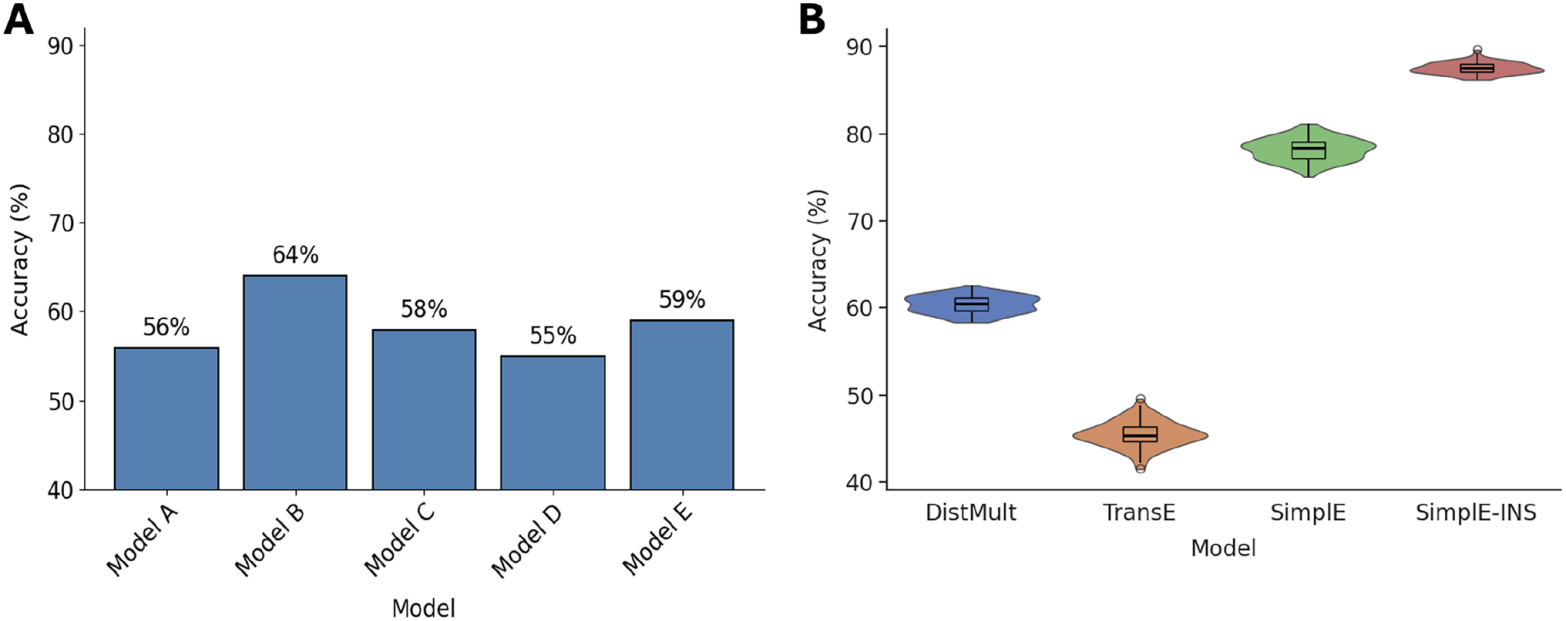
**A** Performance comparison between series of increasingly constrained null models on the test set. Each null model captures specific structural or statistical biases in the data, such as global class frequency, environment-specific tendencies, or strain-level interaction patterns. **B** Comparison of the accuracies of four KGE methods on the test set for predicting pairwise interactions. SimplE and SimplE-INS represent the same model, differing in the choice of the negative sampling strategy.

We further analyzed how each strain behaves when acting as a receiver (Fig. 3C) or sender (Fig. 3D). In both cases, negative interactions dominate for all strains, with some exceptions. For example, *Escherichia coli* (EC), when acting as a sender, shows more positive (313) than negative (260) interactions. Another example is *Pantoea allii* (PAl) strain, which, as a receiver, was involved in 390 neutral interactions, 267 negatives and 99 positive interactions. Based on this, we defined two additional baselines: the receiver strain baseline (Null Model C) and the sender strain baseline (Null Model D), which predict the interaction type in the test set based on the most frequent class involving a given receiver or sender in the training set. Lastly, to evaluate whether the interaction types are consistent across all carbon source environments for a given pair of strains, we computed the entropy of class distribution for each pair of strains. Figure 3E shows the entropy values for each pair of strains, indicating how diverse the interaction types are across all environments when considering the same interacting pair of strains. Most pairs showed high entropy, indicating diverse interaction types rather than one dominant class. However, some exceptions exist, for example, *Raoultella planticola* (RP1) strain when acting as sender and *Enterobacter ludwigii* (EL) as receiver had low entropy, suggesting that a single interaction type tends to dominate. Based on this, we created a pair-specific baseline (Null Model E), which predicts the majority interaction type for each (sender, receiver) pair across environments. Figure 4A shows the performance of these null models. The best model was the environment-specific baseline (Null Model B), achieving an accuracy of $64\%$.

### Prediction of pairwise microbial interactions

To assess the predictive capability of KGE models, we formulated the problem as a link prediction task in a KG. We constructed the KG based on microbial strain interactions, the carbon source environments in which they occur, and the interaction types (Positive, Negative, or Neutral). As shown in Fig. 1A, each triple in the KG represents a pairwise interaction between two species in a given carbon source environment. It consists of three pieces of information: (1) the head, the sender species of the interaction, (2) the tail, the receiver or affected species, and (3) the relation (edge), representing the environment in which the interaction occurs and the type of the interaction. In total, the KG was composed of 20 nodes, 113 relations and consists of 15, 074 triples. This KG consisted solely of microbial interactions, without any additional information. Given two strains and a specific carbon source environment, the main objective was to accurately predict the interaction type. To this end, we evaluate the performance of three KGE models. The prediction of the interaction type between a pair of species was performed by selecting the interaction with the highest score assigned by the model. Fig. 4B depicts the performance accuracy of each model on the test set. The TransE model achieved an accuracy of $0.46 \pm 0.01$, which was lower than all the baseline models (Fig. 4A). The DistMult model achieved an accuracy of $0.60 \pm 0.01$, outperforming the null models A, C, D and E but had a slightly lower accuracy compared to the environment-specific baseline (Null Model B). The SimplE model was the most accurate, with an accuracy of $0.78 \pm 0.01$, outperforming all other models, including all the baseline models.

The choice of the negative sampling (NS) strategy is crucial and significantly impacts the training of KGE models^45,46^. In order to improve the performance of the SimplE model, we implemented an NS method that consists of corrupting the relation component of triples. The comparison between this approach (SimplE-INS) and the classical NS method (SimplE) on the test set is shown in Fig. 4A. The model’s accuracy increased significantly from $0.78 \pm 0.01$ to $0.88 \pm 0.01$ ($P < 0.0001$), confirming the effectiveness of the new method. Therefore, SimplE-INS was selected as the final model.

**Fig. 5:**
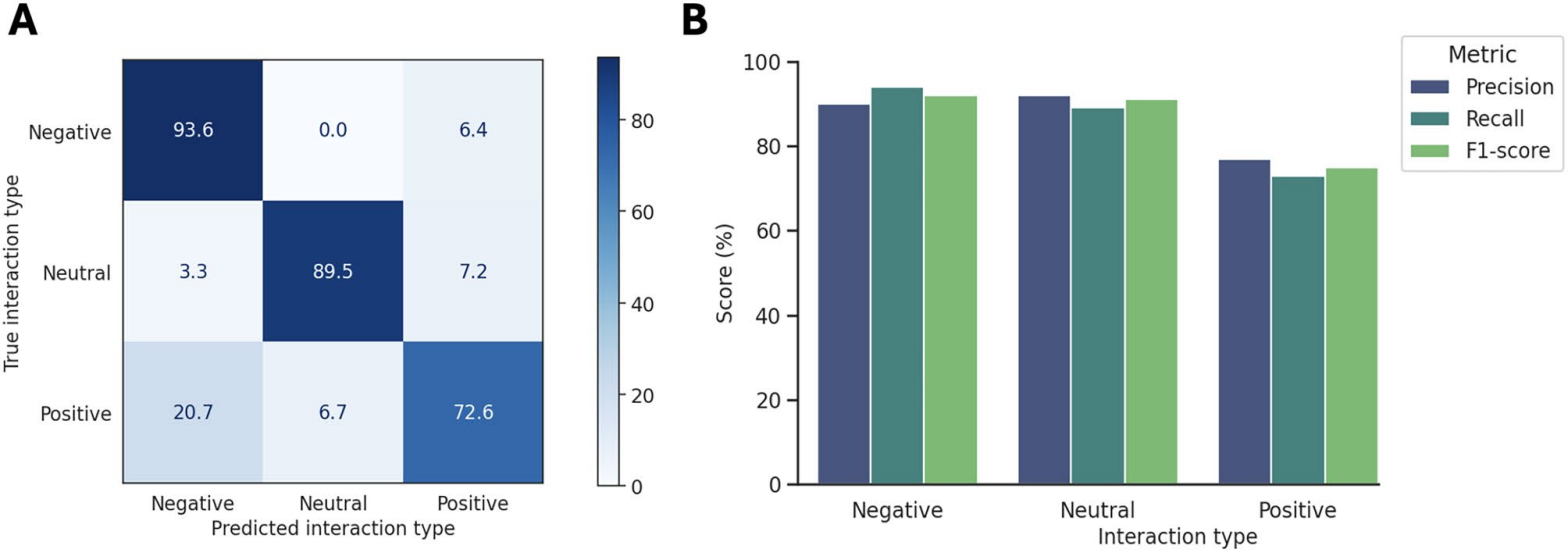
**A** Confusion matrix illustrating the classification performance of SimplE-INS on the test set for predicting pairwise interactions between microorganisms. **B** Precision, Recall and F1-score for each of the three classes, with all values presented as percentages.

Despite the class imbalance, the model achieved high performance across all classes, as shown in the confusion matrix (Fig. 5A). Out of 423 negative interactions (the majority class), the model accurately predicted 396 instances ($93.6\%$), and among 152 neutral interactions, 136 ($89.5\%$) were correctly classified. Additionally, the model correctly classified 130 positive interactions ($72.6\%$) out of 179 instances in the test set. Recall, precision and F1-score are shown in Fig. 5B, demonstrating strong performance for all interaction types.

Lastly, the model was also evaluated using link prediction metrics. It achieved an MRR of 0.87 and a Hits@1 of 0.79, which demonstrates its high performance in accurately ranking triples within the KG, compared to a random baseline model that uses initialized embeddings without training and achieves an MRR of 0.44 and a Hits@1 of 0.24.

**Fig. 6:**
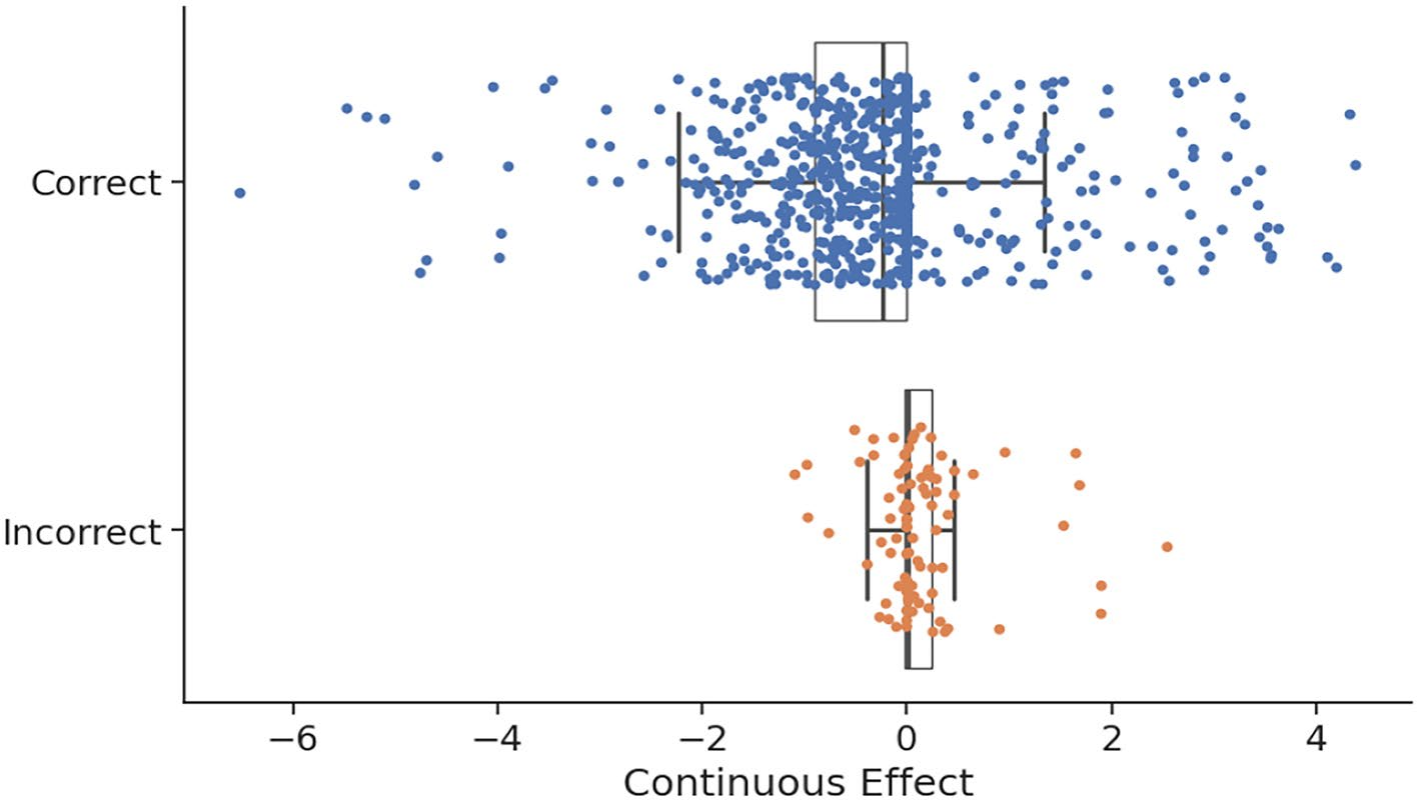
Relationship between prediction accuracy and interaction strength (continuous effect). Each dot represents an interaction from the test set, plotted according to its continuous effect value $E_{S_2 \rightarrow S_1}$ (Eq. 1). Blue dots indicate correctly classified interactions, while orange dots correspond to misclassified ones. The figure shows that most errors occur when the effect is near zero, suggesting lower prediction confidence for weak interactions.

Furthermore, to analyze the model’s predictions and identify its strengths and weaknesses, we compared correct and incorrect predictions with respect to the continuous effect defined in Eq. 1 (Fig. 6). The analysis revealed that incorrect predictions ($12\%$) occurred particularly for interactions with effects near zero. These results indicate that the model is slightly less accurate in classifying some interactions with small effects.

### Predicting interactions for unseen receiver-environment pairs

To further evaluate the generalization capability of our approach, we considered a realistic and challenging scenario in which the model must predict how a target strain responds to other strains in a given environment, without having observed any prior interactions involving that strain as a receiver in that environment. Specifically, we excluded all interactions involving five randomly selected strains when they act as receivers in any environment, which resulted in a test set of 3, 745 triples.

In this setting, since the interaction effect (Eq. 1) depends on both coculture and monoculture growth in a given environment, excluding all triples where a strain acts as a receiver means that the model has no access to any growth data for those strains in any environment. The model was retrained on the remaining data and evaluated on this held-out set.

**Fig. 7:**
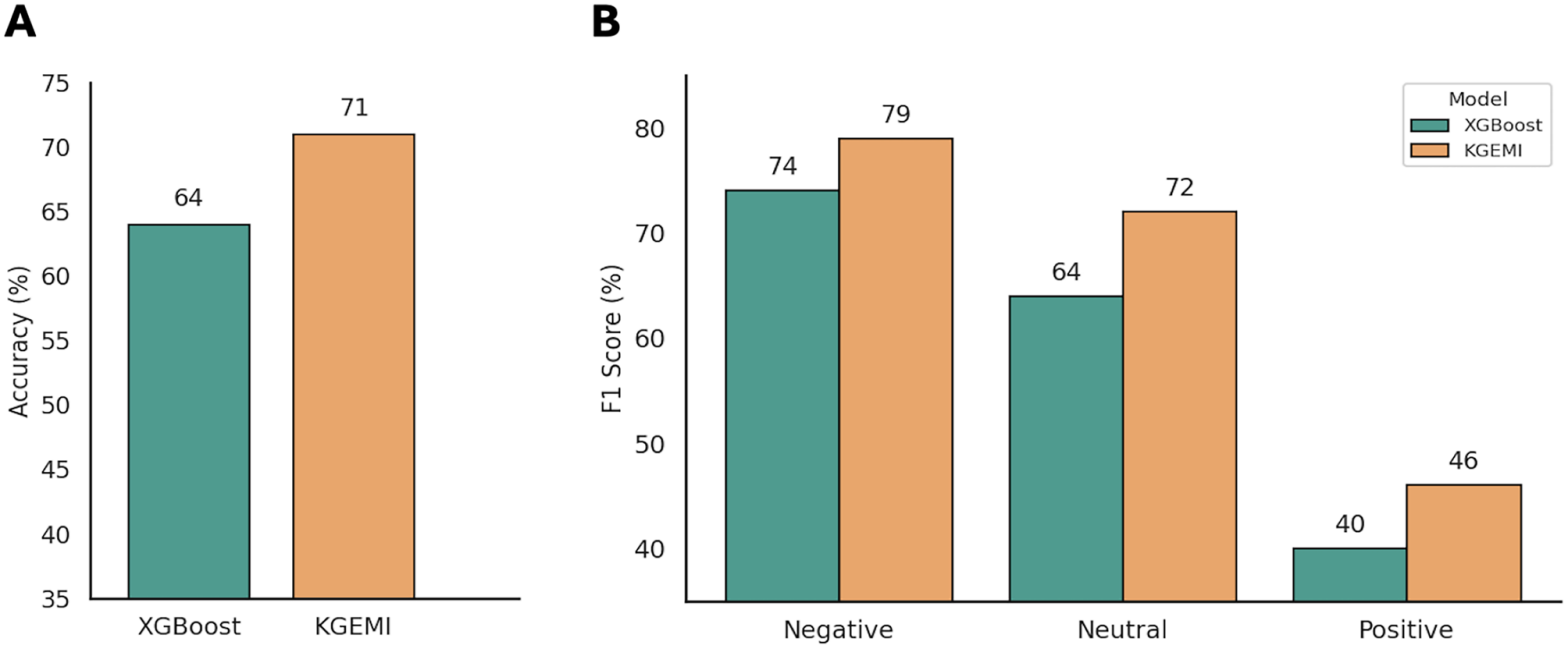
Performance comparison between KGEMI and XGBoost on the task of predicting interactions for unseen (receiver, environment) combinations. **A** Overall accuracy of both models. **B** F1 scores for each model, illustrating how each approach handles the prediction of positive, negative, and neutral interactions when the receiver strain and environment context were not seen during training.

To benchmark performance, we also trained and evaluated an XGBoost classifier under the same conditions. As shown in Fig. 7A, KGEMI outperformed XGBoost with an accuracy of $71\%$ compared to $64\%$, and achieved higher F1 scores across all interaction types (Fig. 7B).

### Strain embedding analysis

It is well accepted that strains with close metabolic profiles tend to be phylogenetically related. However, it is not clear whether this also applies to biotic interactive profiles of bacteria. To investigate the relationship between biotic interaction properties and phylogenetic proximity, t-distributed stochastic neighbor embedding (t-SNE)^47^ was employed for dimensionality reduction, and hierarchical clustering was performed to examine the distribution of the strains within the embedding space. As each microbial strain is represented by two embeddings, we concatenated them to form a single vector representation per strain.

**Fig. 8:**
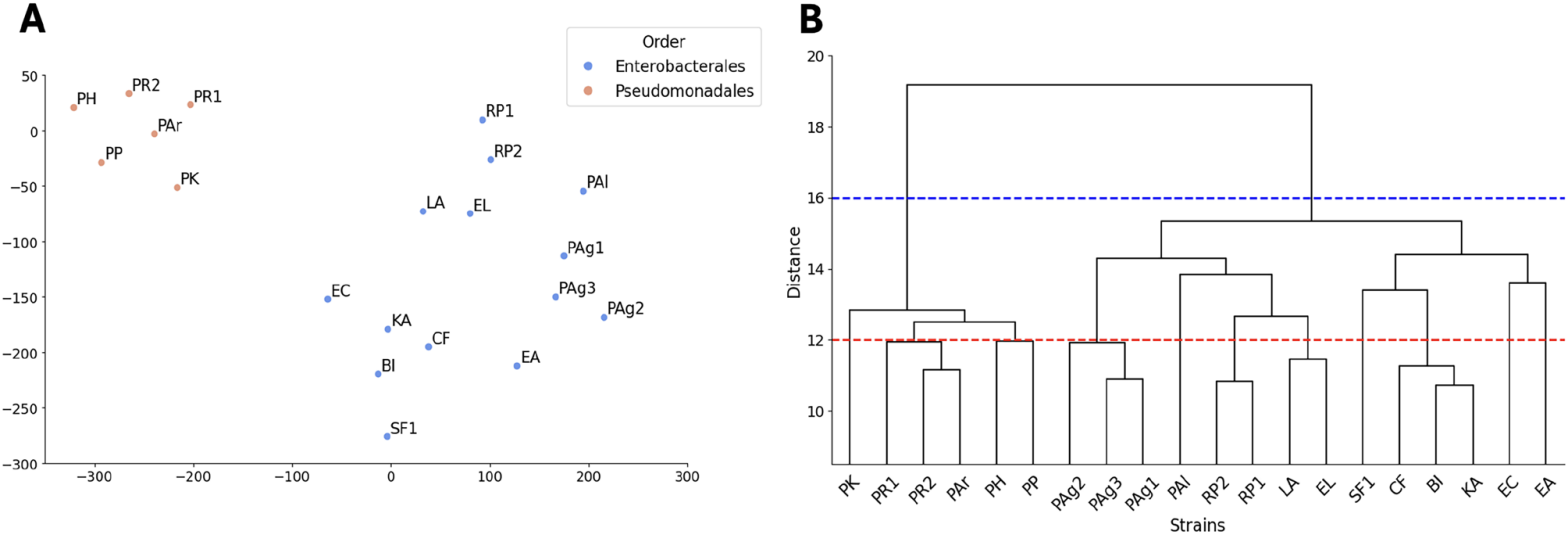
**A** t-SNE visualization of microbial strain embeddings showing the distribution of strains in the embedding space, colored according to their taxonomic orders. **B** Hierarchical clustering dendrogram representing the 20 strains based on their embeddings. The blue dotted line indicates the taxonomic order level, while the red line represents the species level of microbial strains.

Although phylogenetic information was not used during the model training, the similarities between microbial strain embeddings reflected their phylogenetic relatedness. As illustrated in Fig. 8A, microorganisms from the same taxonomic order (Enterobacterales or Pseudomonadales) were grouped together. More importantly, the analysis of the dendrogram (Fig. 8B) revealed that hierarchical clustering based on distances between strain embeddings provided biologically meaningful clusters. For example, a distance threshold of $d = 16$ resulted in two clusters corresponding exactly to the two taxonomic orders. Additionally, the clusters identified with a threshold of $d = 12$ represent, to some extent, the species level of microorganisms. For instance, strains belonging to the same species, such as *Raoultella planticola* (RP1, RP2) and *Pantoea agglomerans* (PAg1, PAg2, PAg3), form the same cluster at $d = 12$. The same observation applies to *Pseudomonas rhodesiae* strains (PR1, PR2). However, for this specific species, one strain from another species, *Pseudomonas arsenicoxydans* (PAr), was found within the same cluster. Overall, these results demonstrate that the model captures taxonomic relationships, suggesting that microorganisms with similar biotic interaction profiles tend to be phylogenetically related.

### Environment-based decision rule for predicting interactions

Analyzing the environment embeddings can facilitate the identification of environments with similar ecological characteristics. Since for each environment there are three relations, each corresponding to one of the three interaction types, we concatenated their embeddings to create a single vector representation of each environment. Consequently, using this vector, we were able to calculate the similarities between different environments and identified the most similar ones.

**Fig. 9:**
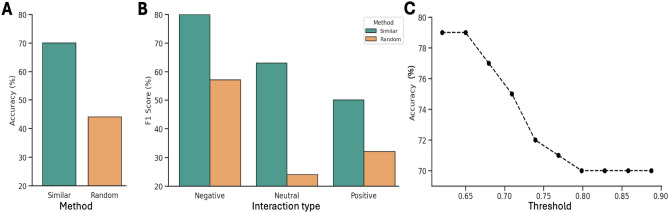
**A** Comparison of overall accuracy between the similarity-based decision rule and the random method. **B** F1-score for each class comparing the performance of both methods. **C** Accuracy of the similarity-based decision rule as a function of the distance threshold. For each threshold, the accuracy was calculated using only the interactions from the test set that occurred in environments with a distance to the most similar environment below that threshold.

We hypothesized that if two environments are highly similar, then the same cocultured strains would exhibit similar ecological behaviors in both environments. Thus, if this assumption is validated, it could serve as a decision rule to infer the interaction type between pair of strains in one environment based solely on the observed outcome in a similar one. To evaluate the predictive power of this rule, we employed it to predict the interaction types in the test set using the information from the training. For each pair of strains in the test set, we predicted the interaction type by assigning the interaction type observed in the most similar environment from the training set between the same pair of strains. This approach achieved an accuracy of 0.70 on the test set. Furthermore, to confirm that this performance was due to the embedding-based similarities between environments rather than chance, we compared it with a random decision rule that consists of predicting the interaction type in an environment based on the outcome in another environment sampled randomly between the same pair of strains regardless of the environmental similarities. As shown in Fig. 9A, the random decision rule achieved a very low accuracy of 0.44, and for all classes the similarity-based decision rule outperformed it (Fig. 9B). This demonstrates that the vector representations of the environments are meaningful and capture the underlying ecological roles that the environment plays in the context of microbial interactions.

We further analyzed the impact of applying a distance threshold when selecting the closest environment. For each threshold, we calculated the accuracy only for environments with a distance below the threshold and excluded the others. As shown in Fig. 9C, as expected, the accuracy decreased as the distance threshold increased. This indicates that the predictions are more reliable for closer environments. Overall, these results demonstrate that this approach can serve as a decision rule to infer the interaction type between a pair of strains in an environment based solely on the observed outcome in a similar one.

### Predicting the interaction outcome for unseen strains using phylogenetic relatedness

The model was not primarily designed to predict interactions for strains that were not seen during the training process. To address this limitation, we implemented a new strategy that takes advantage of the relationship between the embeddings and the taxonomy of microbial strains. As previously highlighted, strains that are closely related phylogenetically have more similar representations in the embedding space. Based on this observation, we created a synthetic embedding to represent the new strain in the embedding space, and used it to make predictions through the model’s scoring function. As described in Fig. 2, for a target strain with missing cultivation data, we created its embedding by aggregating the embeddings of the *k* most phylogenetically related strains from the dataset. This resulted in a synthetic embedding that we considered as representative of the target strain.

**Fig. 10:**
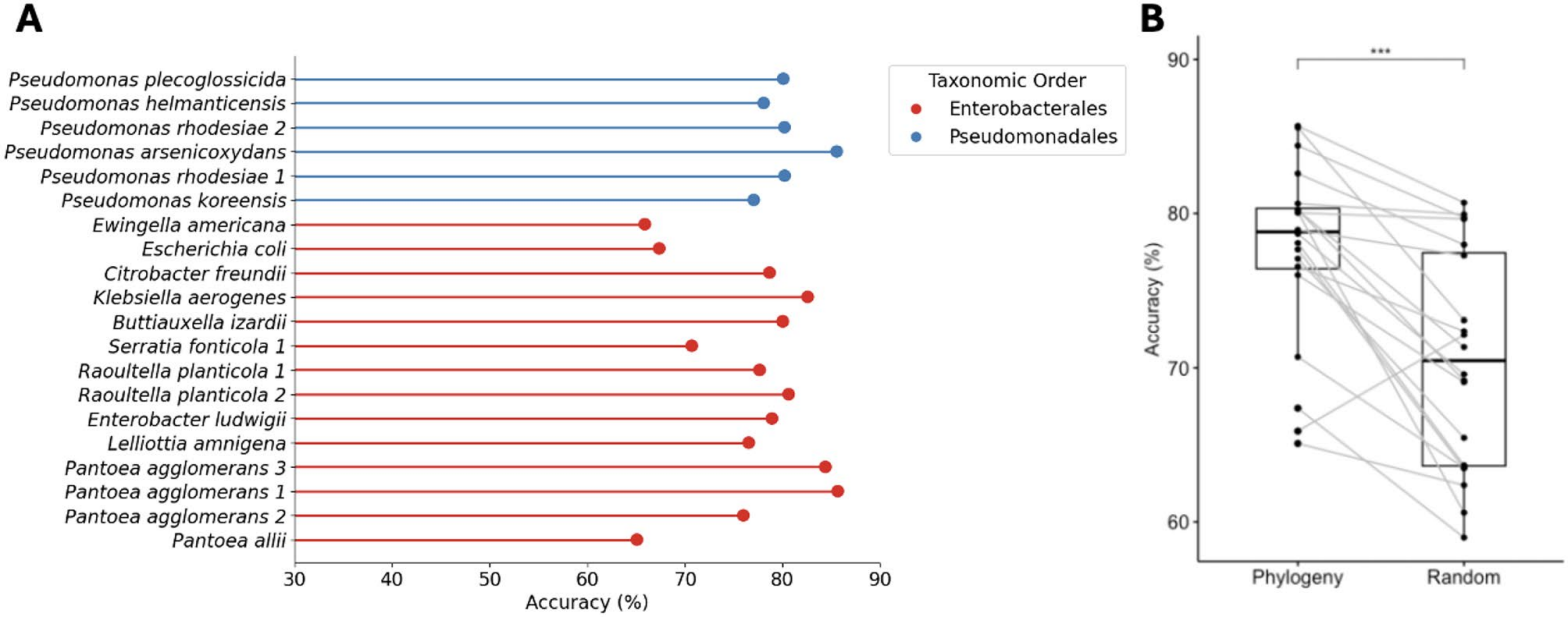
**A** Prediction accuracy of the model for each microbial strain. Each strain was considered as a new (unseen) strain by excluding all its interactions from the training set and keeping them in the test set. **B** Performance comparison between the phylogenetic distance-based and the random methods. Asterisks indicate the significance of the statistical test.

To evaluate this approach, the model was retrained 20 times, once for each of the 20 strains in the dataset. For each target strain, we excluded all its interactions from the training set and kept them in the test set. As there were 6 strains belonging to the order Pseudomonadales and 14 for Enterobacterales, we chose $k = 5$ as a maximum value ensuring that the target strain and its related strains would always belong to the same order. Each resulting model was used to predict the interactions between the target strain and the 19 others in the dataset. Figure 10 shows the results of this approach for each of the twenty strains. The mean accuracy was $0.78 \pm 0.06$. Except for three Enterobacterales species, *Pantoea allii*, *Escherichia coli* and *Ewingella americana*, the performance accuracy was higher than $70\%$.

To further quantify the contribution of phylogenetic information to the model accuracy, we compared the results with another approach that does not rely on phylogenetic relatedness. In this method, we simply generated the synthetic embedding of a strain by averaging the embeddings of $k=5$ randomly selected strains. Using the same evaluation protocol, this method achieved an average accuracy of $0.71 \pm 0.07$ on the test set. Thus, the use of phylogeny significantly improved ($P<0.0001$, Wilcoxon signed-rank test) the accuracy by $7\%$. The detailed accuracies and F1-scores for each strain are provided in Supplementary Table S2. These results show that the model can be used to predict the interaction type between microorganisms that were not used to train the model by leveraging phylogenetic information.

We also conducted the same experiment with different values of $k \in \{2, 3, 4\}$ to assess whether the number of nearest phylogenetic neighbors affects the prediction accuracy. The resulting average accuracies were $0.77 \pm 0.07$ for $k = 2$, $0.77 \pm 0.07$ for $k = 3$, and $0.78 \pm 0.07$ for $k = 4$. Given the small differences and variability between runs (due to random initialization), no clear trend was observed in this range, suggesting that the model performance remains relatively stable for *k* between 2 and 5.

### Case studies: strain recommendations by KGEMI

**Fig. 11:**
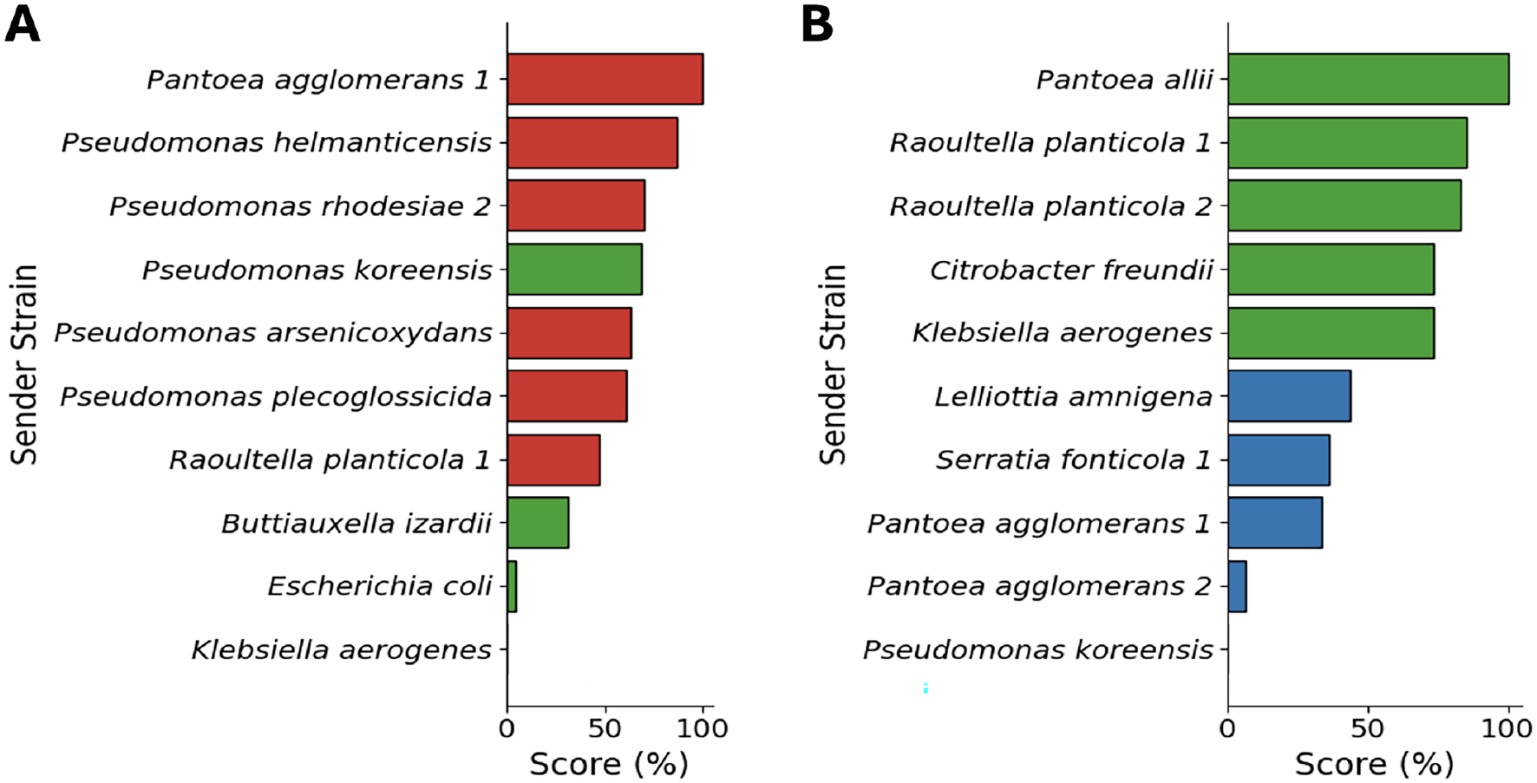
Ranking of strains based on KGEMI’s predicted interaction scores. **A** Predicted score of 10 candidate strains that inhibit *Enterobacter ludwigii* in a sucrose environment. **B** Predicted score of candidate strains promoting the growth of *Pseudomonas arsenicoxydans* in a lactose environment. In both cases, the scores are normalized to facilitate the comparison. Bar colors indicate the actual interaction type from the test set: red for negative, green for positive, and blue for neutral.

Besides predicting microbial interspecies interactions, KGEMI can recommend strains that are likely to inhibit or promote the growth of a target strain, or have no effect on it in a specific environment based on a query. Given an input of candidate strains, KGEMI ranks them in a descending order of likelihood to answer the query. To illustrate the recommendation process of the system, we carried out case studies on two examples of strains: *Enterobacter ludwigii* and *Pseudomonas arsenicoxydans*. In both cases, the interactions involving the target strain and 10 candidate strains were excluded from the training set, while all other interactions, including those unrelated to the target strains, were used during the training process. In the first example (Fig. 11A), the aim was to identify which strain is most likely to negatively impact *Enterobacter ludwigii* in a sucrose environment. Among the 10 candidates, 6 are known to be inhibiting the growth of *Enterobacter ludwigii* (red) from the test set, while the 4 remaining candidates have a known positive impact on the target (green). The top ranked candidates are the most likely to inhibit the growth of *Enterobacter ludwigii* strain in a sucrose environment according to the model. Apart from *Pseudomonas koreensis*, all the three other candidates that are known to have a positive impact had a relatively low score. In the second example, we focused on the strains that are most likely to enhance the growth of *Pseudomonas arsenicoxydans* in a lactose environment. As shown in Fig. 11B, *Pantoea allii* had the highest score, followed by two *Raoultella planticola* strains, *Citrobacter freundii* and *Klebsiella aerogenes* with relatively high scores. All these strains have a known positive effect (green) on the target from the test set, while the effects of the remaining five candidates are known to be neutral (blue).

Since the model was trained as a classification model, its output scores represent the plausibility of a candidate belonging to a specific interaction class (e.g., Positive), and not a continuous estimate of interaction strength. Therefore, the predicted scores are not expected to correlate with the continuous effect values. This analysis is not intended as a formal evaluation of predictive accuracy, as the model was already validated before, instead, it serves to illustrate how KGEMI can be used as a recommendation system to prioritize strains that are likely to lead to a desired interaction with a target strain in a specific environment. The exact ranking of candidates may vary slightly between runs due to random initialization and the small number of candidates considered in each case.

## Discussion

In this article, we presented KGEMI as a multipurpose tool for modeling and predicting microbial interactions, taking advantage of a knowledge graph-based representation that captures the complex relationships between microorganisms. We proposed a knowledge graph-based approach to model microbial interactions, and tested the ability of knowledge graph embedding techniques to predict such interactions. The results demonstrated the strong ability of KGEMI to predict pairwise interactions between microorganisms, achieving an accuracy of $88\%$. The analysis of the generated embeddings enabled us to develop a new technique allowing the prediction of interactions on the basis of phylogenetic relatedness. Furthermore, we built an effective decision rule that uses observed interactions in one environment to infer the interaction outcomes in a similar one. In addition, we have illustrated the capabilities of KGEMI in performing multiple tasks beyond interspecies prediction, such as recommending strains for a predefined ecological engineering purpose, thereby demonstrating that KGE is a promising approach in microbial ecology.

KGEMI significantly reduces the need for input features, requiring only the outcome of interactions between a pair of microorganisms. This is particularly due to the ability of KGE to capture the similarities among microorganisms in the graph as well as their complex relationships across diverse environments. In contrast, existing machine learning-based methods rely on phenotypic and genomic data.

For example, Nestor et al.^23^ developed a method based on XGBoost that has achieved similar performance in predicting pairwise interactions. However, the predictive ability of their approach depends strongly on the availability of the monoculture growth yield of the strains, thus limiting its application to microorganisms for which such information is available. On the other hand, the KGEMI approach described here does not require monoculture growth data to make predictions. To demonstrate this, we tested the model in a setting where five strains were held out as receivers in all environments, simulating a scenario in which growth data is unavailable. KGEMI still achieved strong performance with an accuracy of $71\%$, outperforming XGBoost that achieved an accuracy of $64\%$, thus demonstrating its robustness even when key experimental observations are missing. These two modeling approaches can thus be seen as complementary: depending on the type and availability of input data, either the approach developed by Nestor et al.^23^ or the KGEMI framework can be employed to predict microbial interactions.

Another related machine learning-based study^24^ employed Random forest for interaction prediction based on trait vector representations that encode the presence or the absence of microbial characteristics such as genes and metabolic functions. While this approach has proven strong predictive ability, it was restricted to a single environment. However, environmental contexts are known to significantly impact the outcome of microbial interactions^48,49^. In contrast, thanks to its flexibility in representing the data, KGEMI can be used in both single and multiple environmental contexts.

A major challenge in microbial ecology is the lack of data^50^, which complicates the training of machine learning models, particularly complex ones. To address this, developing simple decision rule models that can predict interactions from limited data is essential. Here, we proposed a simple decision rule model using the similarities based on the embedding of environments. According to this rule, the interaction in an environment is predicted as the interaction outcome from another similar environment between the same pair of competitors. In other words, the way microorganisms interact in an environment can be informative of their interaction in another environment. This approach offers two key advantages. First it enables microbiologists to draw conclusions from minimal information about microbial strains, making it easy to use for practical applications. Second, by identifying environments where microorganisms are likely to exhibit similar ecological responses, it could provide a novel perspective for analyzing microbial communities.

Another advantage of KGE methods over classical machine learning methods is their ability to generate embeddings representing microorganisms and their interactions. We analyzed these vector representations and found that strains that are close phylogenetically had more similar embeddings compared to those that were less related. This indicates that overall, closely related strains are more likely to exhibit similar ecological roles. These results are consistent with the idea that microbial interactions tend to be conserved among closely related strains^51^. Furthermore, based on this, we developed a predictive method enabling interaction prediction even for strains with unknown cultivation data. Specifically, we found that incorporating phylogenetic relatedness information contributed to a significant $7\%$ increase in accuracy. This is consistent with a previous study^23^ showing that a phylogenetic model had some predictive power to predict the interactions. It is worth noting that our approach is general and not limited to phylogenetic information. Other factors such as growth conditions, when available, can also be incorporated to generate synthetic embeddings for unseen strains using the same process. Moreover, we propose a KGE-based recommendation system designed to suggest strains that are more likely to inhibit or promote the growth of a target strain, or to have no effect on it. Such systems could assist microbiologists in understanding the interactions between microorganisms and thereby improve microbial community engineering.

Since classical machine learning-based methods operate only on tabular data, KGE models can help extract useful topological patterns from a network and incorporate them as features to train a machine learning model. For example, in a recent study^52^, KGE was combined with a protein language model to train XGBoost to predict phage-host interactions. Additionally, to leverage the generated embeddings, one could also use them in clustering tasks to analyze communities and gain insights into microbial interactions^53^.

In conclusion, this study demonstrates the effectiveness of KGE techniques in predicting microbial interactions while requiring minimal input features. It offers a new perspective on modeling pairwise interactions, as it highlights not only KGE’s ability to capture the complex structure of microbial interactions but also the importance of analyzing the generated embeddings. Future studies should investigate the advantage of constructing knowledge graphs with more diverse information (e.g., genetic, metabolic, environmental factors). Such extensions could enhance prediction accuracy and broaden the scope of the model to include additional microbial traits, such as optimal growth conditions, thereby positioning it as a more general framework beyond pairwise interactions. Furthermore, the predictive power of a KGE model depends strongly on the representation of knowledge in the KG. Here, we represented the microorganisms as nodes and their relationships encoded the interaction types and carbon source environments. However, exploring other possible knowledge representations could lead to both better predictive ability and analysis. A key limitation of this study is that it relies on a relatively small set of bacterial strains with limited phylogenetic diversity, all of which are cultivable under laboratory conditions. As the vast majority of microbial species cannot be grown in vitro^54^, this restricts the direct applicability of the current approach to more complex or natural microbial communities. Future extensions may incorporate metagenomic or co-occurrence data to enable predictions in broader ecological contexts, including uncultured species. While we used coculture interaction data, the design of our approach is flexible and can, in principle, incorporate other types of relational data. For example, sequencing-based data such as co-occurrence networks could be used to construct a knowledge graph in which microbial taxa are nodes and edges represent patterns of co-occurrence or mutual exclusion. Environmental contexts could be incorporated either as relation types, as in our current implementation, or as additional edge features, allowing the model to learn how environmental factors shape microbial interactions.

Lastly, while pairwise interaction outcomes can have some predictive power for predicting survival in high-order competitions^19^, future work should aim to evaluate KGE on more complex communities. One possible direction would be to construct a knowledge graph for each microbial community, where nodes represent microbial taxa and edges encode contextual information such as environmental factors, known interactions, or co-occurrence patterns. This problem can be seen as graph-level task, in which the model takes a whole graph as input and predicts either a diversity index or the composition of the community. While this approach is promising, its feasibility depends on the availability of high-quality community-level dataset that include both the structure of each community and environmental conditions.

## Supplementary Information

Supplementary Information.

## Data Availability

The code and all data are available at https://github.com/Medkne/kgemi
