# Supplementary material for: Knowledge graph embedding for predicting and analyzing microbial interactions
Source: Sci Rep. 2025 Nov 21;15:43541. doi: 10.1038/s41598-025-27591-9 (PMC12695954; doi:10.1038/s41598-025-27591-9)
Supplement: Supplementary file 1 — Supplementary Information. [file 41598_2025_27591_MOESM1_ESM.pdf]

Supplementary data

October 17, 2025

Table S1: Taxonomic classification and closest species matches for the 20 microbial strains used in this study.

**Strain shorthand**	**Order**	**Closest match**
EA	Enterobacterales	*Ewingella americana*
RP1	Enterobacterales	*Raoultella planticola*
BI	Enterobacterales	*Buttiauxella izardii*
CF	Enterobacterales	*Citrobacter freundii*
PAg1	Enterobacterales	*Pantoea agglomerans*
KA	Enterobacterales	*Klebsiella aerogenes*
RP2	Enterobacterales	*Raoultella planticola*
PAg2	Enterobacterales	*Pantoea agglomerans*
SF1	Enterobacterales	*Serratia fonticola*
LA	Enterobacterales	*Lelliottia amnigena*
PAl	Enterobacterales	*Pantoea allii*
PAg3	Enterobacterales	*Pantoea agglomerans*
EL	Enterobacterales	*Enterobacter ludwigii*
EC	Enterobacterales	*Escherichia coli*
PH	Pseudomonadales	*Pseudomonas helmanticensis*
PR1	Pseudomonadales	*Pseudomonas rhodesiae*
PP	Pseudomonadales	*Pseudomonas plecoglossicida*
PR2	Pseudomonadales	*Pseudomonas rhodesiae*
PK	Pseudomonadales	*Pseudomonas koreensis*
PAr	Pseudomonadales	*Pseudomonas arsenicoxydans*

Table S2: F1-scores for each interaction class (Negative, Neutral, Positive) and overall accuracy for phylogeny-based and random baseline predictions across microbial strains.

**Strain**	**Phylogeny**				**Random**			
	**Negative**	**Neutral**	**Positive**	**Accuracy**	**Negative**	**Neutral**	**Positive**	**Accuracy**
PAl	0.77516576	0.50933333	0.46800731	0.65087957	0.75292588	0.5235732	0.39542484	0.62381597
PAg2	0.8430055	0.77011494	0.53978159	0.76	0.82422105	0.71610169	0.51674641	0.69071429
PAg1	0.91195652	0.85863874	0.67379679	0.85675857	0.87014428	0.76947536	0.47882136	0.80699395
PAg3	0.90332975	0.8409894	0.65	0.84404284	0.88996243	0.81362007	0.62081129	0.79718876
LA	0.85667752	0.68619247	0.56481481	0.76549865	0.77212389	0.51340996	0.34169279	0.72371968
EL	0.87880351	0.72611465	0.54576271	0.78933333	0.85744345	0.70517928	0.49246231	0.77266667
RP2	0.88937093	0.73893805	0.59375	0.80640669	0.83243824	0.5010989	0.34234234	0.79944290
RP1	0.85929919	0.72727273	0.57450077	0.7766463	0.82650197	0.63888889	0.50529501	0.63475900
SF1	0.78994614	0.67419962	0.53781513	0.70713306	0.78908189	0.70588235	0.54545455	0.63511660
BI	0.87715517	0.732	0.62857143	0.80040188	0.81584821	0.71280277	0.43181818	0.79638312
KA	0.90401786	0.75446429	0.6516129	0.82587413	0.86605081	0.72043011	0.5754386	0.77972028
CF	0.87672747	0.67266187	0.616179	0.7868296	0.88036117	0.76656151	0.63703704	0.69585879
EC	0.75605214	0.76811594	0.42530283	0.67377839	0.75185185	0.77272727	0.40896359	0.58981418
EA	0.7650463	0.66666667	0.39492243	0.65896739	0.78723404	0.71886121	0.4057971	0.72146739
PK	0.84720639	0.79859895	0.54185693	0.77062374	0.80090243	0.65232975	0.38402458	0.65459423
PR1	0.86283705	0.82097649	0.63407407	0.80231766	0.83870968	0.79857398	0.56586826	0.69188821
PAr	0.90097933	0.89943074	0.68680445	0.85571142	0.8010929	0.66789668	0.41800643	0.73079492
PR2	0.85842194	0.85384615	0.61870504	0.80213191	0.81968967	0.6064257	0.38425197	0.71352432
PH	0.85224586	0.80232558	0.58258258	0.78079332	0.79450487	0.62172285	0.35750422	0.63674322
PP	0.84382567	0.91531532	0.61872456	0.80095109	0.7081571	0.60218978	0.28070175	0.60597826
